# Knockdown of Rab21 inhibits proliferation and induces apoptosis in human glioma cells

**DOI:** 10.1186/s11658-017-0062-0

**Published:** 2017-12-19

**Authors:** Jian Ge, Qianxue Chen, Baohui Liu, Long Wang, Shenqi Zhang, Baowei Ji

**Affiliations:** Department of Neurosurgery, Renmin Hospital of Wuhan University, No.9 Zhangzhidong Road, Wuchang District, Wuhan, Hubei 430060 People’s Republic of China

**Keywords:** Glioma, Rab21, RabGTPases, Proliferation, Apoptosis

## Abstract

**Background:**

Gliomas are commonly malignant tumors that arise in the human central nervous system and have a low overall five-year survival rate. Previous studies reported that several members of Rab GTPase family are involved in the development of glioma, and abnormal expression of Rab small GTPases is known to cause aberrant tumor cell behavior. In this study, we characterized the roles of Rab21 (Rab GTPase 21), a member of Rab GTPase family, in glioma cells.

**Methods:**

The study involved downregulation of Rab21 in two glioma cell lines (T98G and U87) through transfection with specific-siRNA. Experiments using the MTT assay, cell cycle analysis, apoptosis assay, real-time PCR and western blot were performed to establish the expression levels of related genes.

**Results:**

The results show that downregulation of Rab21 can significantly inhibit cell growth and remarkably induce cell apoptosis in T98G and U87 cell lines. Silencing Rab21 resulted in significantly increased expression of apoptosis-related proteins (caspase7, Bim and Bax) in glioma cells.

**Conclusions:**

We inferred that Rab21 silencing can induce apoptosis and inhibit proliferation in human glioma cells, indicating that Rab21 might act as an oncogene and serve as a novel target for glioma therapy.

## Background

Gliomas, which are the most frequent malignant primary tumors in the central nervous system (CNS), have a survival rate of less than 10% [1–3]. The common treatment for a glioma is surgical resection and radiotherapy. Due to the highly infiltrative features of these tumor cells, it is very difficult to remove all of them via surgical resection, particularly in eloquent areas of the brain [4]. While chemotherapy and radiotherapy can kill most tumor cells, those that survive typically regrow. Identifying and characterize offing molecular regulators that relate to glioma oncogenesis and development of glioma might inform future therapeutic strategies [5–7].

Several molecular regulators of cancer development have been identified recently, including Rab small GTPases (Rabs). Previous studies demonstrated that Rabs play important roles in various cellular functions, including growth, development and membrane trafficking [8–11]. Rabs interact with a lot of effector and affector proteins involved in membrane fusion and directed transport of organelles along cytoskeletal frameworks.

In human malignancies, including glioma, abnormal expression of Rabsled to aberrant tumor cell behavior. Rab23 is implicated as a metastatic or tumorigenic biomarker as it shows differential expression in tumor cells and has been demonstrated to mediate proliferation and invasiveness in tumor cells [12]. Overexpression of Rab3a or Rab27a in glioma cells can both promote cell proliferation and inhibit cell apoptosis [13, 14]. Rab38 is a newly discovered member of the Rab family. Its expression is closely correlated with grade progression and prognosis in gliomata [15]. RabGTPase 21 (Rab21) was originally cloned from the canine MDCKII cell library, but its expression has been reported as ubiquitous [16, 17]. Rab21 is a member of the Rab subfamily, which has been identified as required for tumor-associated fibroblasts to promote the invasion of squamous carcinoma cells. However, no functional Rab21 data have been reported in gliomata.

In this study, we characterize the roles of a novel member of Rab subfamily, Rab21 in glioma. We found that a significant increase occurred in Rab21 expression in T98G and U87 glioma cell lines and that downregulation of Rab21 using specific-siRNA transfected with two glioma cell lines (T98G and U87) significantly inhibits cell growth and remarkably induces cell apoptosis. It was demonstrated that Rab21 might work as an oncogene and serve as a novel target for glioma therapy.

## Methods

### Cell culture and RNA interference

Human glioma cell lines U251, T98G, SHG44, U87 and U373 were obtained from the Cell Bank of Chinese Academy of Sciences. They were cultured in a 5% CO_2_/95% humidified air atmosphere at 37 °C in Dulbecco’s modified Eagle’s medium (DMEM; Gibco) supplemented with 10% fetal bovine serum (FBS; Hyclone). The siRNA targeting human Rab21 (siRab21, 5’-GGCCAGGATTTCAAATCCA-3′) and nonspecific siRNA (siNC) were purchased from GenePharma Company. siRNAs or siNC were inserted into U87 and T98G cells using Lipofactamine 2000 (Invitrogen) according to the manufacturer’s instructions. Assays were performed 48 h after transfection.

### RNA extraction and quantitative real-timePCR

Total RNA was extracted from samples usingTrizol reagent (TaKaRa) and the concentration wasdetected with an ultraviolet spectrophotometer. PrimeScriptRT Reagent Kit with gDNA Eraser (TaKaRa) was used to obtain cDNA according tothe manufacturer’s protocols.

The total volumesfor PCR were 20 μl,containing 10 μl of 2 × SYBR Premix Ex TaqTM (TaKaRa), 0.50 μmol/l forward primer,0.50 μmol/l reverse primer and 0.2 ± 0.02 μg cDNA template. The reaction conditions were pre-denaturation at 95 °C for 30 s, followed by 40 cycles of denaturation at 95 °C for 5 s, annealing at 60 °C for 20 s and elongation at 72 °C for 20 s.

The mRNA levels of the aim genes were calculated using the ΔΔCt method. β-actin was used as the housekeeping gene. Three samples were detected and three technical replicates were measured for all groups.

### Western blotting

Protein expression levels were analyzed via western blot. Cells were washed twice with PBS and lysed with RIPA buffer. The protein concentration was detected using a BCA Kit (Beyotime). Equal amounts of protein (30 μg) were separated using 10% SDS-PAGE gel and transferred onto PVDF membranes (Millipore). After blocking with 5% skim milk for 2 h at room temperature, the membranes were incubated with primary antibodies overnight at 4 °C.

The specific primary antibodies were rabbit anti-Rab21 (Abcam; 1:1000 dilution), rabbit anti-Caspase7 (Abcam; 1:500 dilution), rabbit anti-Bim (Abcam; 1:500 dilution), rabbit anti-Bax (Santa Cruz Biotechnology, 1:200 dilution) and mouse anti-actin (Cell Signaling Technology; 1:1500 dilution).

The membranes were then washed with Tris-buffered and incubated with corresponding horseradish peroxidase-conjugated secondary antibodies (Beyotime; 1:1000 dilution) for 1 h at room temperature. Immunoreactivity was visualized using a Millipore Enhanced Chemiluminescence system. Membranes were scanned with a Bio-rad Gel Doz EZ imager.

### Cell proliferation assay

Cell proliferation was evaluated using the MTT colorimetric method (3-(4,5-dimethylthiazolyl-2)-2,5-diphenyltetrazolium bromide; Sigma-Aldrich).The cells were placed in 96-well plates at a concentration of 1 × 10^4^/well and transfected with siRab21 or siNC using Lipofectamine 2000 (Invitrogen) according to the manufacturer’s instruction. MTT (20 μl) was added into each well and incubated for 2–4 h at 37 °C (at 0, 24, 48 and 72 h post-transfection). Once the purple precipitate was visible, 150 μl isopropanol and 0.04 mol/l HCl were added and the cells incubated at room temperature in the dark for 2 h. The absorbance was recorded at 570 nm. The full procedure was repeated 3 times.

### Cell cycle assay

At 48 hpost-transfection, the cells were trypsinized, washed three times with PBS, collected, and fixed with 70% ice-cold ethanol overnight at 4 °C. Then propidium iodide (PI) was added for staining, and the cells were incubated for 30 min at 4 °C in the dark. Finally, flow cytometry (BD FACScaliber) was used to evaluate the percentages of cells in each phase, including G0/G1 phase, S phase and G2/M phase.

### Cell apoptosis assay

Apoptosis was analyzed with a BD AnnexinV-FITC/PI flow cytometry kit according to the manufacturer’s instructions. At 48 h post-transfection, the cells were collected and washed with ice-cold PBS three times and resuspended in 200 μl of binding buffer at a concentration of 1 × 106 cells/ml. 10 μl Annexin V-FITC and 10 μl PI were added and the cells were incubated for 30 min at 4 °C in the dark. Finally, 300 μl binding buffer was added and analyzed by flow cytometry (Beckman Coulter, Cytomics FC 500) within 1 h.

### Statistical analysis

The SPSS 18.0 software package was used for the statistical analyses. Each experiment was repeated at least three times and all data are expressed asmeans ± SD. Dunnettʼs one-way analysis of variance (ANOVA) was used to evaluate the difference in the results. *p* < 0.05 was considered statistically significant.

## Results

### Expression of Rab21in human glioma cell lines

**Fig. 1:**
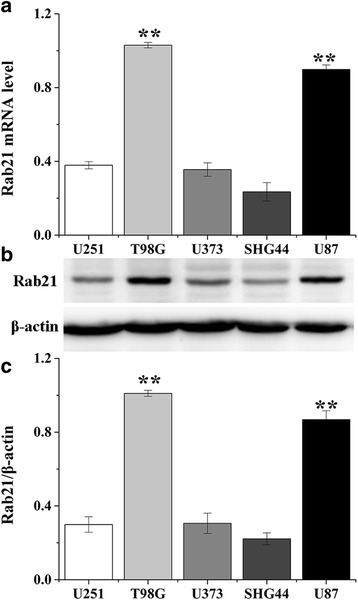
Rab21 expression in human glioma cells. The mRNA (**a**) and protein (**b** and **c**) levels of Rab21 in five glioma cell lines were determined via qRT-PCR and western blot, respectively. **P<0.05 compared with U251 cell lines

Rab21 expressionsin terms of mRNA and protein levels were evaluated in five human glioma cell lines: U251, T98G, U373, SHG44 and U87. T98G and U87 cells exhibited higher levels of Rab21expression (Fig. 1).

### Downregulation of Rab21 in human glioma cells by siRNA transfection

**Fig. 2:**
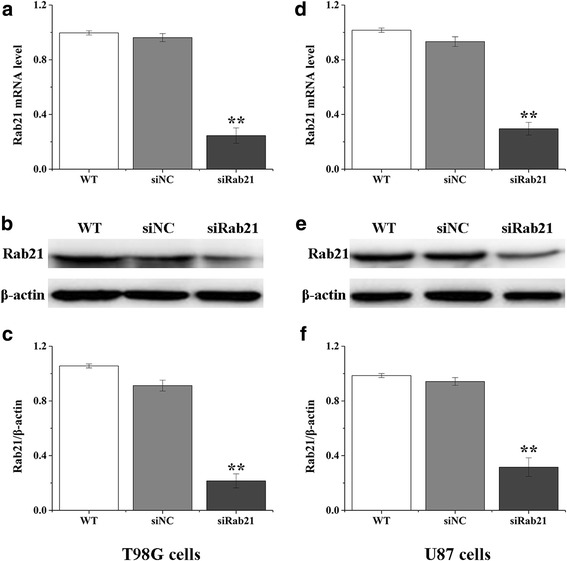
Knockdown of Rab21 in glioma cells. Downregulation of Rab21mRNA and protein expression in T98G (**a**–**c**) and U87 (**d**–**f**) cells by Rab21siRNA was determined using RT-PCR and western blot, respectively. **p < 0.01 compared with the siNC group

To further evaluate the effect of Rab21in glioma cells, Rab21was knocked down by transfection with synthetic human Rab21 siRNA (siRab21) in U87 and T98G cells. The mRNA and protein expression levels of Rab21 were significantly reduced by Rab21 siRNA transfection in both cell lines (Fig. 2).

### Rab21knockdown inhibits glioma cell proliferation

**Fig. 3:**
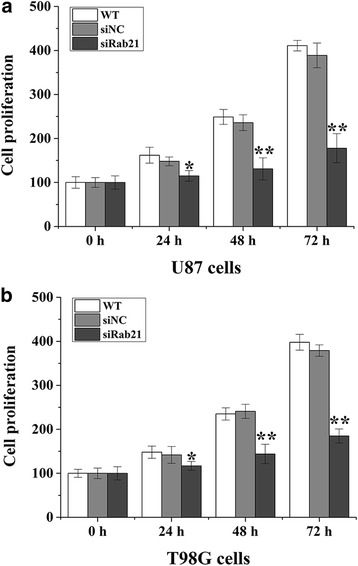
Rab21knockdown inhibits glioma cell proliferation. Human Rab21siRNA were transfected into T98G (**a**) and U87 (**b**) cells, followed by cell proliferation determination using the CCK-8 assay. The results of cell proliferation were normalized to the initial cell numbers (100%). *p < 0.05,**p <0.01 compared with the siNC group

To determine the detailed effect of silencing Rab21 on glioma cells, the proliferations of U87 and T98G cells were measured using the MTT assay at 0, 24, 48 and 72 h post-transfection. The cell proliferation in the Rab21 siRNA-transfected group (siRab21) had decreased significantly compared with that in the WT group and siNC groups (Fig. 3). No significant difference was observed in the proliferation between the WT and siNC group (*p* > 0.05).

### Rab21knockdown in glioma cells induces cell cycle arrest in the G0/G1 phase

**Fig. 4:**
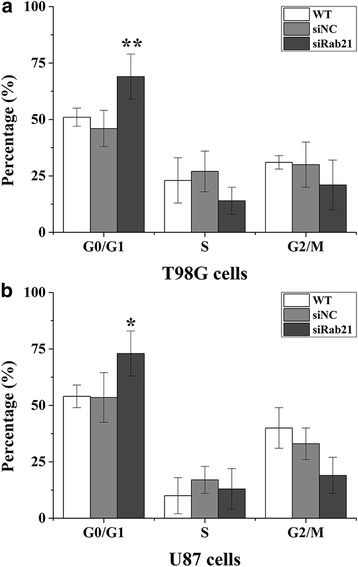
Effect of Rab21 siRNA on cell cycle distribution in glioma cells. After transfection with Rab21 siRNA or Rab21-specific siRNA for 48 h, the cell cycle distribution of T98G (**a**) and U87 (**b**) cells was determined via flow cytometry. Representative images of cell cycle distribution alterations and cell percentages in different cell cycle phases after Rab21 knockdown are shown. *p < 0.05, **p < 0.01 compared with siNC group

PI staining was used to detect the cell cycle phase distributions in glioma cells 48 h after Rab21 siRNA transfection. As shown in Fig. 4, both U87 and T98G cells were remarkably arrested in G0/G1 phase after Rab21 knockdown. The numbers of cells in S phase and G2/M phase had also decreased, but there was no statistical difference.

### Silencing Rab21induces cell apoptosis in human glioma cells

**Fig. 5:**
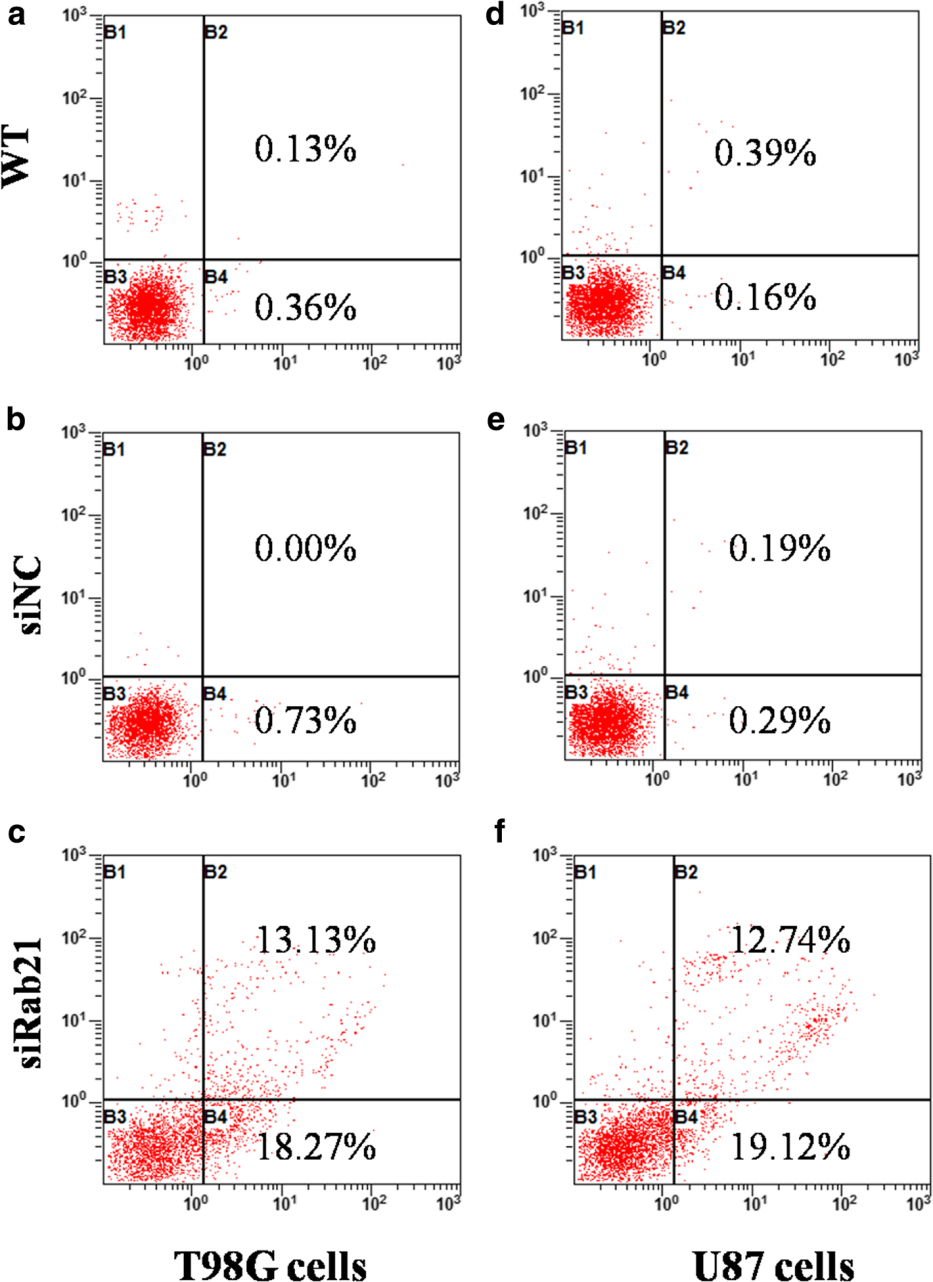
Rab21 knockdown induces cell apoptosis in glioma cells. After transfection with Rab21siRNA or Rab21-specific siRNA for 48 h, the cell apoptosis of T98G (**a**–**c**) and U87 (**d**–**f**) cells was assessed via flow cytometry analysis

Cell apoptosis was detected usingPI/Annexin V staining followed by flow cytometry. As shown in Fig. 5, the levels of early and advanced stage apoptosis had increased significantly in both U87 and T98G cell lines transfected with Rab21 siRNA.

**Fig. 6:**
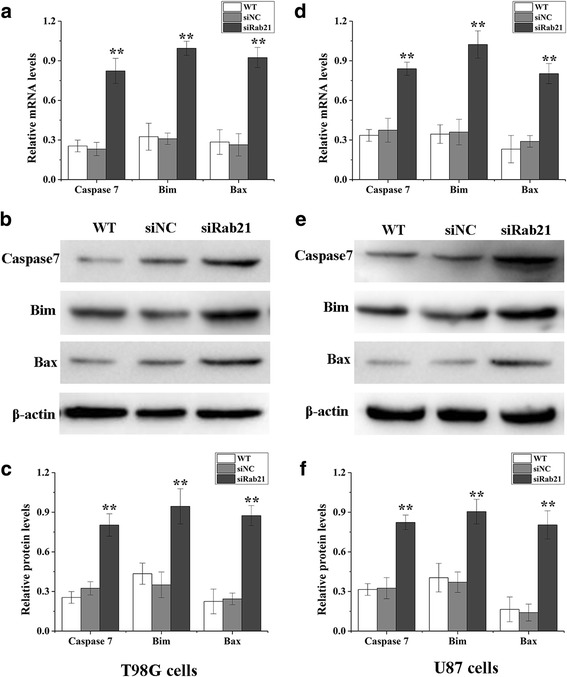
Effects of Rab21 siRNA on the expression of apoptosis-related proteins. After transfection with Rab21 siRNA for 48 h in T98G (**a**–**c**) and U87 (**d**–**f**) cells, the mRNA (**a**, **d**) and protein (**b**, **c**, **e** and **f**) levels of apoptosis-related proteins were determined using qRT-PCR and western blot, respectively

The expression levels of apoptosis-associated proteins were measured using qRT-PCR and western blot. As shown in Fig. 6, knockdown of Rab21by siRNA induced the expression of the apoptotic proteins, caspase7, Bax and Bim, the levels of which had increased significantly in U87 and T98G cells.

## Discussion

Gliomata, which is one of the most common cancers of the CNS, has a very low overall five-year survival rate [2, 3]. Identifying and characterizing novel molecular biomarkers or regulators related to its oncogenesis and development is essential in the advancement of therapeutic options for glioma.

Previous studies demonstrated that several Rab small GTPases are involved in glioma oncogenesis, playing important roles in its prognosis [12, 14]. In this study, we suggested a tumorigenic effect of Rab21 in the development of glioma. We found that downregulation of Rab21 by specific siRNA inserted in two glioma cell lines (T98G and U87) significantly inhibited cell proliferation and remarkably induced cell apoptosis.

Here, we describe the first functional characterization of the small GTPaseRab21 in glioma. The disorder expression of Rab GTPase has been implicated in various cancers [18, 19]. The expression of Rab21 was reported to regulate the adhesion and migration of several tumor cell types, including breast, prostate and liver cancer [20].

In this study, Rab21 was shown to have a higher expression in glioma cell linesT98G and U87. It was previously reported that Rabs expression changes could lead to abnormal trafficking of the growth factor receptors, cell surface integrins and matrix metalloproteinases, which could further increase cell proliferation and migration [21].Our results show that the proliferation of T98G and U87 cells decreased significantly and that the apoptosis of T98G and U87 cells was induced when Rab21 was downregulated. The expressions of the apoptosis-related proteins caspase7, Bim and Bax were increased significantly in T98G and U87 cells upon Rab21 downregulation.

The investigation of apoptosis in tumors will both give an insight into the pathogenesis of tumors and provide clues for tumor treatment. It is well known that caspase 7 is one of the key downstream effectors in p53-dependent apoptosis and that Bim and Bcl-2 are pro-apoptosis proteins [22–24]. The phosphorylation of Bim by JNK1/2 has shown to increase its binding affinity to Bcl-2, which translates into a better activation of Bax/Bak with subsequent cytochrome c release and caspase7 activation [25, 26]. Caspase7 acts as an activating “effector” caspase with short domains that in turn cleave intracellular substrates leading to the dramatic morphological and biochemical changes of apoptosis [27]. In this study, the mRNA and protein expressions of caspase7, Bim and Bax increased significantly in T98G and U87 cells transfected with the siRab21 vector. We inferred that this essential apoptosis-regulating pathway is activated in gliomata after Rab21 knockdown. Our findings show that silencing Rab21 exerts anti-tumor effects by altering apoptosis-related protein expressions in glioma cells.

## Conclusions

Our results strongly indicate that knockdown of Rab21 in glioma cell lines T98G and U87 results in a decrease in cell proliferation via cell cycle arrest and subsequent induction of apoptosis. Consequently, we suggested that Rab21 might serve as a biomarker for prognosis and a potential target for glioma therapy.

## Abbreviations

CNS: central nervous system
PI: propidium iodide
Rab21: RabGTPase 21
